# A Novel Bionic Catalyst-Mediated Drug Delivery System for Enhanced Sonodynamic Therapy

**DOI:** 10.3389/fbioe.2021.699737

**Published:** 2021-07-30

**Authors:** Yiling Yang, Shaohua Hua, Weilong Suo, Wenbin Wang, Longhao Wang, Zhengguang Chen, Kefeng Liu, Jie Zhao

**Affiliations:** ^1^Department of Ultrasound, The First Affiliated Hospital of Zhengzhou University, Zhengzhou, China; ^2^Key Laboratory of Applied Chemistry and Nanotechnology at Universities of Jilin Province, Changchun University of Science and Technology, Changchun, China; ^3^Academy of Medical Sciences, Zhengzhou University, Zhengzhou, China; ^4^Department of Pharmacy, The First Affiliated Hospital of Zhengzhou University, Zhengzhou, China; ^5^Internet Medical and System Applications of National Engineering Laboratory, Zhengzhou, China

**Keywords:** sonodynamic therapy, curcumin, hollowed MnO_2_, catalyst, tumor cell membrane

## Abstract

Ultrasound (US)-triggered sonodynamic therapy (SDT) proves itself to be a formidable tool in the fight against cancer, due to its large spectrum of uses as a non-invasive therapeutic measure, while also demonstrating itself to be a certain improvement upon traditional SDT therapeutics. However, tumor hypoxia remains to be a major challenge for oxygen-dependent SDT. This study describes the development of an innovative, multi-use, catalyst-based and improved SDT targeting cancer, through the employment of a sonosensitizing curcumin (Cur) load embedded within a MnO_2_ core, together with an extraneous tumor cell membrane component. The latter allows for efficient tumor recognition properties. Hollowed-out MnO_2_ allows for efficient drug delivery, together with catalyzing oxygen generation from hydrogen peroxide present in tumor tissue, leading to enhanced SDT efficacy through the induction of a reduced hypoxic state within the tumor. In addition, Cur acts as a cytotoxic agent in its own right. The results deriving from *in vivo* studies revealed that such a biomimetic approach for drug-delivery actually led to a reduced hypoxic state within tumor tissue and a raised tumor-inhibitory effect within mouse models. Such a therapeutic measure attained a synergic SDT-based tumor sensitization treatment option, together with the potential use of such catalysis-based therapeutic formulations in other medical conditions having hypoxic states.

## Introduction

Breast cancer remains a major cancer condition afflicting woman on a global scale, with present therapeutic measure still not providing full patient recoveries, particularly in metastatic phases, which typically lead to a 90% mortality rate (Ho, 2020). However, during this new millennium, theragnostic nanomedical approaches could provide novel treatment options in oncology (Zhang et al., 2019; Zhu D.M. et al., 2020; Zhu et al., 2021). The advantages stemming from such novel technologies include temporal and/or spatial regulation, surgical-like accuracy in tumor detection and a marked reduction in cytotoxicity (Jin et al., 2020; Suo et al., 2020; Zhu Y. et al., 2020). The last 10 years have seen the research community focusing on light-mediated photodynamic therapy (PDT) (Zhang et al., 2016; Ren et al., 2020; Suo et al., 2020; Zhu Y. et al., 2020), where light-induced sensitizer elicitation leads to tumor cell apoptosis through the up-regulated secretion of reactive oxygen species (ROS), which are highly cytotoxic (Meng et al., 2019; Hu et al., 2020; Li S. et al., 2020). However, one major issue with PDT is the light penetrative capacity, whereby deep-seated tumors are less susceptible to such a therapeutic measure (Li C. et al., 2020; Ding et al., 2021). In addition, a reduced half-life of less than 0.04 μs (Xu et al., 2020), together with elevated ROS reactiveness allows for a therapeutic effect to be functional for a penetrative depth of less than 0.02 μm (Huang et al., 2021). Conversely, SDT demonstrates itself as an advancement over PDT due to its enhanced tissue penetrative properties, non-invasive nature and reduced costings.

Presently, SDT is hindered from acting as a novel therapeutic option due to its reduced release of ROS in hypoxic states (Huang C. et al., 2020). In this respect, multiple research efforts focusing on angles such as enhanced direct-delivery of oxygen (O_2_) and hydro-photolysis are still limited in efficacy. This is due to issues of sub-standardized tumor capillary networks (Liu et al., 2017), reduced O_2_-generation efficacy (Meng et al., 2018; Huang C. et al., 2020), together with premature O_2_ leaks (Li et al., 2017; Zhang et al., 2017; Chen et al., 2018). Consequently, novel alternatives are required for tumor oxygen-content “terraforming,” in order to enhance SDT efficacy.

As a substance which speeds up a chemical reaction, a catalyst is supposed to have a high-performance specificity to promote product generation (Fan et al., 2015; Huang J. et al., 2020). Catalase can catalyze the breakdown of hydrogen peroxide and thus improve the tumor microenvironment (Xu et al., 2019; Zinger et al., 2019). Notwithstanding, naturally-occurring catalysts are costly and highly challenging to be successfully delivered within tumor microenvironments (Qin et al., 2018; Lei et al., 2019; Qin et al., 2020). Consequently, current research efforts are focusing on developing artificial catalysts bearing the same potentials of naturally-orrurring catalase. Innovative MnO_2_ nanoparticles (NPs) act as catalase in low-pH tumor microenvironments, leading to a reduced hypoxic state (Yang et al., 2017). A recent multi-use hollowed-out MnO_2_ nanocatalyst successfully served its objectives to reduce hypoxic states and as a drug-delivery system (Lyu et al., 2020). Once introduced, this nanocatalyst accumulates within the kidneys and is eventually cleared out as Mn^2+^ with minimal cytotoxicity effects (Zhao et al., 2014; Huang J. et al., 2020). However, no previous investigations of the effectiveness of such NPs in sensitizing SDT were performed.

**SCHEME1:**
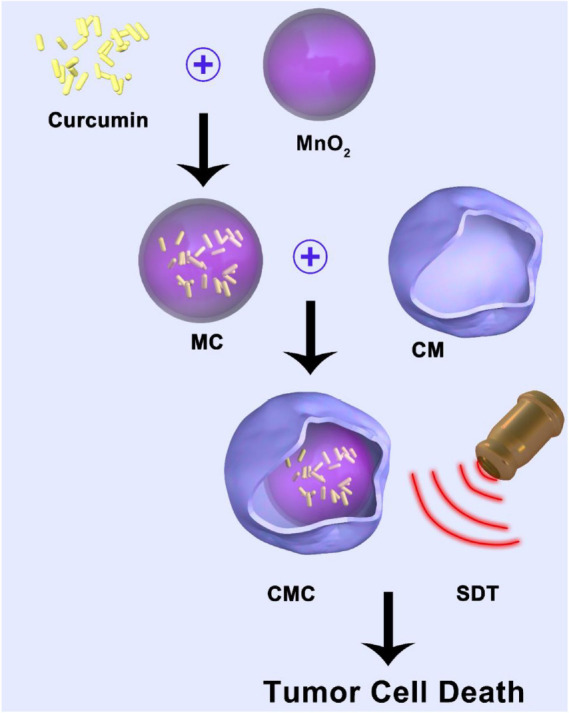
Biomimetic catalyst hybrid formulation for synergic-induced sonodynamic therapy.

This investigation concerned the development of a tumor cell-membrane biomimetic catalyst/curcumin hybrid system (CMC) with an internalized core of curcumin (Cur)-loaded MnO_2_ NPs for the objective of enhancing SDT efficacy (Scheme 1). Curcumin proves itself to be an effective cancer chemotherapy agent through its pro-apoptotic function, together with a sonodynamic influence on the tumor cell (Wang et al., 2013; Ayyanaar et al., 2019). In murine cancer models treated with intravenous CMC, successful delivery into hypoxia-afflicted tumor microenvironments was recorded. The outer layer, consisting of a tumor cell membrane, allowed for enhanced survival time within bloodstream circulation together with enhanced NP accumulation at the targeted tumor site. Once the NP was phagocytosed by the individual tumor cell, the composite formulation was allowed to violently interact with the high-concentrated, low pH environmental hydrogen peroxide, leading to a major oxygen-burst. Concomitantly,MnO_2_ degradation into Mn^2+^ ions facilitated Cur release into the tumor cell cytoplasm, leading to pro-apoptotic and anti-proliferative effects on the tumor cell, while still achieving minimal collateral cytotoxic and adverse effects. Furthermore, Cur also exhibited sonodynamc efficacy on the individual tumor cell. The oxygen-burst was successful in redicing the hypoxic state within the tumor microenvironment and consequently sensitized such tumor tissues to SDT measures. Both *in vitro* and *in vivo* confirmed our novel CMC formulation provides a major therapeutic impact on tumor tissues with minimal adverse effects.

## Results and Discussion

**FIGURE 1:**
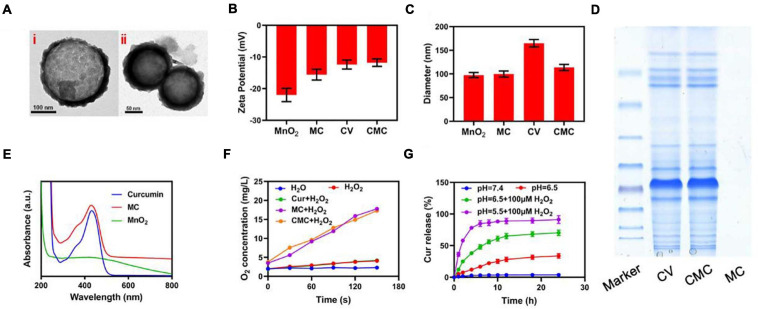
Characterization of CMC. **(A)** TEM images of (i) MC and (ii) CMC. **(B)** Zeta potential and **(C)** Hydrodynamic diameter of MnO_2_ NPs, MC, CV and CMC. **(D)** Coomassie-stained SDS-PA gel image showing the protein composition of CV, CMC and MC. **(E)** Absorbance spectrum of Curcumin, MC and MnO_2_. **(F)** Oxygen generation at different conditions measured using dissolved oxygen meter. **(G)** Cur release from CMC under different conditions.

This study successfully managed to design and synthesize hollowed-out MnO_2_ NPs, together with successful Cur loading through mechanical mixture generation. Regarding the latter process, MnO_2_ NPs (10 mg) were dissolved within Cur solution with continuous stirring in a dark area for 24 h. Consequently, NPs were re-collected through centrifuging procedures. The CMC loading efficacy for Cur was 62.4 ± 4.5%. Transmission electron microscopy (TEM) imaging highlighted that Cur-loaded MnO_2_ NPs (MC) had a mean diameter of 98.5 nm. The tumor cell membrane coated vesicle (CV) MC NPs has the appearance of a 5 nm-girth gray outer-shell when viewed under TEM (Figure 1A). Cur loading procedures and successful CV coat applications were additionally validated through fluorescence localization dynamic light scattering (DLS), SDS-PAGE and UV-Vis spectrometry analyses. Although both MnO_2_ NP and MC formulations attained equivalent diameter sizes, the CMC formulation was marginally larger in size (MnO_2_ NPs = 98.4 ± 5.3 nm; MC = 99.1 ± 3.7 nm; CMC = 109.8 ± 3.4 nm). And We have measured the size distribution of nanoparticles using DLS. As the polydispersity index (PDI) of these nanoparticles are 0.212; 0.226; 0.283 corresponds to MnO_2_, CM, and CMC. Also, as the stability of nanomaterials is very important for biological applications. We evaluated the hydrodynamic diameter of CMC for three consecutive days, and the results showed that our material had good stability (Supplementary Figure 1). This finding suggests success in obtaining NP encapsulation into membrance-coated vesicles (Figure 1C). This was also confirmed through Zeta potential readings for all formulations (Figure 1B). Furthermore, CMC successfully maintained retention of the vast majority of CV protein-content (Figure 1D) and also exhibited its signature Cur peaks at 432 nm (Figure 1E). As highlighted in Figure 1F, neither Cur nor deionized water had any specific influence on hydrogen peroxide, albeit both CMC/MC successfully developed a time-dependent effervescence effect upon contact. Consequently, CMC kept its nanozyme potentials, with Cur/CV additions not affecting the essential catalysis functional roles for CMC. Following from this step, analysis focus on Cur release effectiveness by the CMC formulation, under varying environmental circumstances. As highlighted in Figure 1G, Cur was quickly released by CMC formulation in hydrogen peroxide-rich, low pH (5.5) conditions, while minimal such release was noticed when CMC was placed in PBS solution (no hydrogen peroxide). On comparing sustained drug-release profiles for CMC at neutral pH (7.4), the release/jettison rates were found to be more rapid when CMC was placed in moderately acidic solutions (pH = 6.5), ideally also in the presence of hydrogen peroxide). Such findings suggest that the CMC formulation can be degraded within the tumor environment prior to its uptake by individual tumor cells.

**FIGURE 2:**
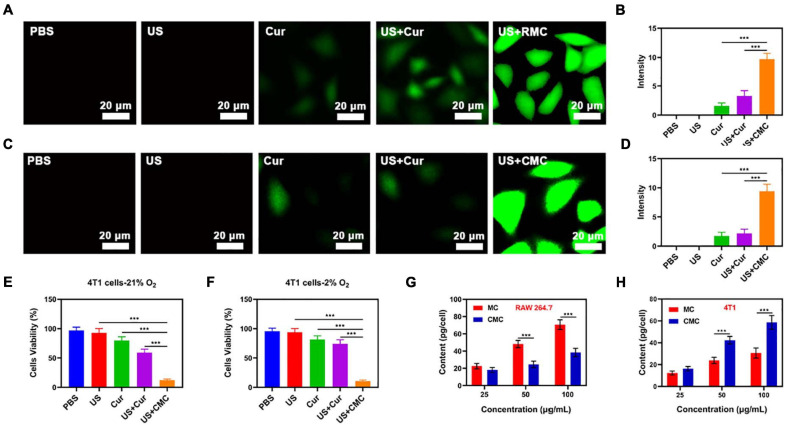
**(A)** Tumor cell fluorescence images and **(B)** DCFH-DA fluorescence intensity following the indicated treatments under normoxia condition. **(C)** Tumor cell fluorescence images and **(D)** DCFH-DA fluorescence intensity following the indicated treatments under hypoxia condition. The survival of HepG2 cells with Control, US, Cur, US + Cur and US + CMC detected by MTT assay under **(E)** 21% O_2_ condition or **(F)** 2% O_2_ condition (*n* = 5). **(G)** Nanoparticle uptake by RAW 264.7 cells at different incubated concentration (MnO_2_ dose of 25, 50, and 100 μg/mL). **(H)** Nanoparticle uptake by HepG2 cells at different concentration. (MnO_2_ dose of 25, 50, and 100 μg/mL). Significance between every two groups was calculated by the Student’s *t*-test. ***P < 0.005.

The fluorescent 2′,7′-dichlorodihydrofluorescein diacetate (DCFH-DA) probe was consequently employed for assessing CMC-directed hydroxyl (-OH) group development within individual tumor cells *in vitro*, under varying experimental conditions. The PBS/US-coated tumor cells exhibited low DCFH fluorescence readouts (see Figures 2A,B), indicating a lack of ROS development within a local microenvironment that has no exogenous treatment components. This finding concurs with the occurrence that tumor cells release high GSH levels in comparison to healthy cells, leading to more effective ROS inhibition. Combinatory treatment involving Cur and US also exhibited a reduced DCFH fluorescence profile, suggesting that internal oxygenation levels, albeit following a combination treatment, were not sufficient for US-facilitated -OH large-scale developments. In addition, low fluorescence was registered for Cur + US treatment study groups placed in hypoxia-laden conditions (Figures 2C,D), suggesting the requirement for adequate oxygenation levels for SDT function. However, US + SDT therapy was found to enhance fluorescence levels within hypoxic states, suggesting enhanced tumor eradication potential.

Consequently, RAW264.7 cell lines were employed for the analysis of immune circumvention potential by MC and CMC NPs, through the concomitant application of inductively coupled plasma-atomic emission spectrometry in order to attain uptake quantification (see Figure 2G). The CMC study group exhibited a significantly reduced uptake, suggesting its effectiveness in evading cell phagocytotic activity, while also displaying high tumor recognition specificity (Figure 2H). Following from such positive findings, CMC-directed anti-tumor adeptness was analyzed through the employment of a cell apoptosis kit. Within non-hypoxic states, elevated cytotoxic function was identified by the Cur + US study group, in comparison to the control study group (Figure 2E). Apoptotic levels peaked within the US + CMC study group, possibly due to the synergic effects of successful immune circumvention by CM, Mn^2+^-catalysis-driven oxidative burst development, together with Cur-based and Cur-mediated sonodynamic roles. In the case of hypoxia, onlythe US+CMC group showed the best therapeutic effect, while thetherapeutic effect of other groups was significantly inhibited(Figure 2F).

**FIGURE 3:**
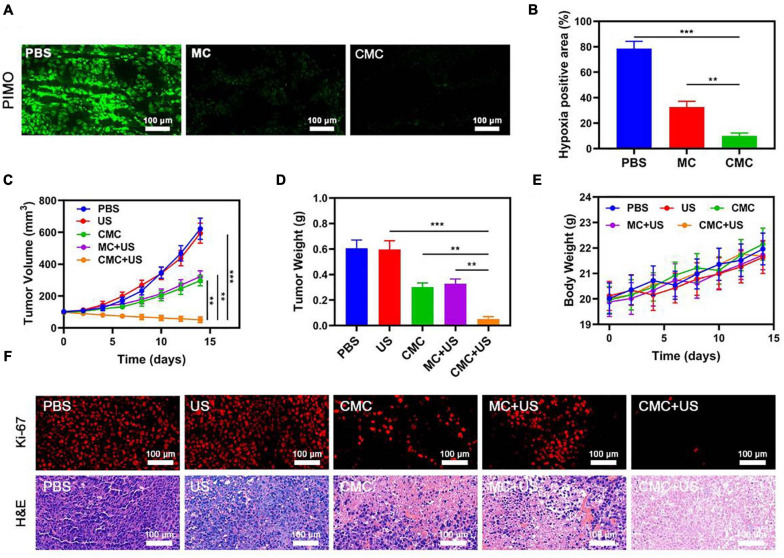
**(A)** Representative images of tumor tissue sections stained with anti-PIMO (green) following the indicated treatments. **(B)** Fraction of pimonidazole (PIMO) positive hypoxic area in different groups (*n* = 3). Changes in tumor **(C)** volume and **(D)** were monitored in mice subjected to the indicated treatments (*n* = 5). **(E)** Changes in murine body weight were monitored over time in the indicated treatment groups (*n* = 5). **(F)** Tumor sections from the indicated treatment groups were subjected to Ki-67 or H&E staining. **P < 0.01, ***P < 0.005; Student’s *t*-test.

A pharmacokinetics assay was consequently performed using CMC for analyzing the outer-coat tumor cell membrane functions on PMS blood-retention. SD murines were treated with intravenous MC or CMC, with a MnO_2_ dosing of 10 mg/Kg (Supplementary Figure 1). The CMC study group demonstrated significantly higher blood-retention properties, consequently suggesting the level of efficacy by the cell membrane outer-coat in extending immune circumvention times for MSNs. Following from this finding, the bio-distribution properties of MnO_2_ within both study groups were scrutinized, with the employment of ICP-MS for measuring manganese content within tumor tissues/organs following intravenous MC or CMC administration. This analysis highlighted that heart and kidney silicon distribution was minimal, though such accumulations took place within the tumor site/s and liver within 24 h post-treatment. Manganese biodistribution demonstrated to be elevated within the CMC study-group, suggesting successful tumor specificity efficacy by the outer-coat tumor cell membrane for this synergic formulation. *In vivo* experiments in hypoxicenvironments also confirmed that our material couldsignificantly relieve hypoxic tumor regions due to its goodtargeting ability (Figure 3A,B).

Owing to the promising tumor-targeting capabilities of such CMC preparations, the *in vivo* CMC anti-tumor activity was consequently investigated. Murines bearing HepG2 tumors were randomly assigned to seven different treatment groups (each group included five murines): (1) PBS; (2) US, 1.0 MHz, 1.5 W/cm^2^, 50% duty cycle, 1 min; (3) CMC; (4) MC + US; (5) CMC + US. The Cur dose was 5 mg/Kg in groups 2, 3, and 4. The SDT was performed 6 h after intravenous injection. The treatment was conducted every 2 days for 14 days. Murine body weight was monitored every 2 days. Tumors in murines within the PBS group rapidly grew to ∼610 mm^3^ in size by day 14. A Cur-equivalent dose of 5 mg/Kg was administered to all murines in the MC and CMC treatment groups. US treatment alone can slightly inhibit tumor growth. Such data suggest that US treatments alone were not potent enough to markedly suppress tumor growth kinetics in this model system. Conversely, tumor growth was markedly suppressed in the CMC and MC + US groups, consistent with the US activity for this preparation. CMC + US treatment exhibited anti-tumor efficacy comparable to that observed in the CMC and MC + US groups. Maximal tumor growth arrest was observed for murines in the CMC + US treatment group, consistent with the ability of CMC to efficiently ablate tumor tissue (Figures 3C,D). No significant body weight changes were observed in treated murines throughout the course of this study (Figure 3E). H&E and Ki-67-stained tissue sections (Figure 3F) from such murines additionally confirmed that CMC + US treatment was linked to significant loss of tumor tissue and was associated with substantial apoptosis-driven necrosis.

**FIGURE 4:**
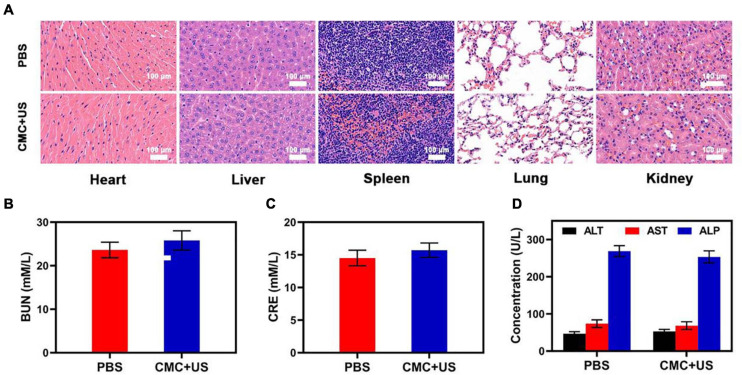
**(A)** Histopathologic examination of the tissues including heart, liver, spleen, lung, and kidney from tumor-bearing mice after PBS or CMC + RT treatment. **(B–D)** Blood biochemistry data of kidney and liver function markers: BUN, CRE, ALT, AST, and ALP.

Based on such findings, this study was able to successfully achieve combination US and CMC treatment, effectively enhancing the tumor killing effect of US and thus increasing the clinical potential of this treatment strategy. Collected major organs from treated mice were utilized to assess CMC-related adverse effects, though no evidence of inflammation or histological abnormalities in any examined samples following CMC treatment were detected (Figure 4). Serum levels of alanine aminotransferase (ALT), aspartate transaminase (AST), alkaline phosphatase (ALP), blood urea nitrogen (BUN), and creatinine (CRE) were also within normal ranges, indicating that renal and hepatic function was normal in such murines (Supplementary Figures 5–7). CMC treatment was therefore not linked to any adverse outcomes in treated murines. These findings suggest that CMC is highly biocompatible and facilitates synergic US treatment, underscoring its value as a promising therapy agent.

## Conclusion

In essence, this study demonstrated the successful design and implementation of CMA as an innovative drug-delivery formulation exploiting a biomimetic tumor cell membrane outer-coating for cloaking effects against immune surveillance within the bloodstream transit and for enhancing tumor specificity for this formulation. Concomitant administration of this formulation with SDT allowed Cur to exert its sonodynamic properties to regulate tumor expansion and through pro-apoptotic mechanisms, stemming from MnO_2_ – induced reduction of hypoxia within the tumor microenvironment. In addition, the CMC-SDT synergic combination therapy was not undermined by major collateral cytotoxicity or adverse effect issues, within multiple assays. Such findings render our novel therapeutic measure to be a potential emerging tool in combating deep-seated tumors with hypoxic states, typically present in cancer patients.

## Data Availability Statement

The raw data supporting the conclusions of this article will be made available by the authors, without undue reservation.

## Ethics Statement

The animal experiments were strictly implemented based on the plan approved and released by the Ministry of Health of China and also approved by the Animal Research Management Committee of Wuhan University.

## Author Contributions

YY and SH: conceptualization. WW: methodology and formal analysis. YY and WS: software and data curation. LW, ZC, and KL: validation. WS: writing—original draft preparation, funding acquisition, and project administration. ZC and WS: writing—review and editing. JZ: visualization, supervision, and resources. All authors have read and agreed to the published version of the manuscript.

## Conflict of Interest

The authors declare that the research was conducted in the absence of any commercial or financial relationships that could be construed as a potential conflict of interest.

## Publisher’s Note

All claims expressed in this article are solely those of the authors and do not necessarily represent those of their affiliated organizations, or those of the publisher, the editors and the reviewers. Any product that may be evaluated in this article, or claim that may be made by its manufacturer, is not guaranteed or endorsed by the publisher.
