# Supplementary material for: A Novel Bionic Catalyst-Mediated Drug Delivery System for Enhanced Sonodynamic Therapy
Source: Front Bioeng Biotechnol. 2021 Jul 30;9:699737. doi: 10.3389/fbioe.2021.699737 (PMC8361452; doi:10.3389/fbioe.2021.699737)
Supplement: Supplementary file 1 [file Table_1.DOC]

**Materials**

Treaethyl orthosilicate (TEOS) and Calcein were purchased from Aladdin Reagent (Shanghai, China). Potassium permanganate (KMnO_4_), and sodium carbonate (Na_2_CO_3_) were obtained from Sinopharm Chemical Reagent CO., Ltd. (China). Phosphate buffer solution (PBS) was obtained from Thermo-Fisher (Waltham, MA, USA). Mito-Tracker Green, 3,3′-dioctadecyloxacarbocyanine perchlorate (DiO), and Reactive Oxygen Species Assay Kit were purchased from Beyotime Company (China). All of the aqueous solutions were prepared using purified deionized (DI) water purified with a purification system (Direct-Q3, Millipore, USA). The other solvents used in this work were purchased from Sinopharm Chemical Reagent (China) and Aladdin-Reagent (China).

**Cell culture and animal models**

HepG2 human liver cancer cell line was obtained from the Cell Bank of the Chinese Academy of Sciences and incubated in RPMI-1640 medium supplemented with 10% FBS in a humidified atmosphere at 37℃. Cell cultures under normoxic conditions (pO_2_: 21%) were maintained in a humidified incubator at 37℃ in 5% CO_2_ and 95% air. Hypoxic conditions (pO_2_: 2%) were produced by placing cells in a hypoxic incubator (Moriguchi, Japan) in a mixture of 2% O_2_, 5% CO_2_, and 93% N_2_.  HepG2 (1 × 10^6^) in 100 μL of PBS in suspension was injected into all mouse bodies in the form of a subcutaneous injection of cells to complete the effective construction of tumor models. When the diameter of the tumor volume reached 200 mm^3^, the tumor-bearing mice were used for the next stage of the experiment. The animal experiments were strictly implemented based on the plan approved and released by the Ministry of Health of China and also approved by the Animal Research Management Committee of Wuhan University.

**Synthesis of hollow MnO_2_ nanoparticles (MnO_2_)**

Solid silica nanoparticles (sSiO_2_) were synthesized following the reported method. Then an aqueous solution of KMnO_4_ (300 mg) was dropwise added into the suspension of sSiO_2_ (40 mg) under ultrasonication. After 6 h, the precipitate was obtained by centrifugation at 14,800 rpm. The as-prepared mesoporous MnO_2_-coated sSiO_2_ was dissolved in 2M Na_2_CO_3_ aqueous solution at 60 °C for 12 h. The obtained hollow mesoporous MnO_2_ nanoparticles (MnO_2_) were centrifuged and washed with water several times.

**Synthesis of curcumin (Cur) loaded MnO_2_ nanoparticles (MC), and cancer cell membranes coated MC nanoparticles (CMC)**

Cancer derived vesicles (PV) amd RBC derived vesicles (RV) were prepared according to the previous work. For Cur loading, MnO_2_ nanoparticles (10 mg) were dispersed in the Cur DMSO saturated solution (5 mg/mL, 6 mL) and kept stirring under dark for 24 h. After that, the nanoparticles were collected by centrifugation (12000 rmp, 25 min) and further dispersed in ultrapure water. To determine the encapsulation efficiency (EE) of Cur, the concentration of unloaded Cur were analyzed by a UV–vis detector. The EE were calculated by using the following formulas: weight of feeding drug—weight of redundant drug / weight of feeding drug) × 100%. For CV coating, the solution of CV (1 mg/mL, 5 mL) and MC (1 mg/mL, 5 mL) were mixed and the mixture was extruded through the 200 nm polycarbonate porous membrane for 11 times by the Avanti mini extruder. And the CMC were isolated by centrifugation.

**Characterization of the CMC nanoparticles.**

The morphology structures of MC, and CMC nanoparticles were observed by the TEM (JEOL-2100). Hydrodynamic diameter and zeta potential were detected by the dynamic light scattering (Nano-ZS ZEN3600). SDS-PAGE was also used to analyze the protein components onto nanoparticles.

**Degradation and drug release studies.**

To study the Cur release, a solution of CMC was dialyzed against PBS with PBS (pH 7.4) or PBS (pH 5.5) with the presence of H_2_O_2_, respectively. The amounts of Cur release at different time points were measured by UV–vis spectra. The morphology structures of CMC nanoparticles were observed by the TEM (JEOL-2100).

**Evaluation the generation of oxygen**

PBS, Cur, MC and CMC (fixed the concentration of Cur with 10 μM) were suspended in 3% H_2_O_2_ solution or ultrapure water (8 mL), respectively. A DOG-3082 oxygen dissolving meter was used to monitor the real-time concentration of oxygen.

***In vitro* immune evasion study**

RAW 264.7 were seeded in 12-well plates and cultured for 12 h. Different concentrations (50, 100 and 200 μg/mL MnO_2_) of MC, and CMC were added the medium. Then the cells were incubated for 2 h at 37 °C, 5% CO_2_, and then washed with PBS three times. The nanoparticles uptake was measured by ICP-MS.

***In vitro* cancer targeting study**

HepG2 cells were seeded in 24-well plates and cultured for 12 h. Different concentrations of MC and CMC were added the medium. Then the cells were incubated for 0.5 h at 37 °C, 5% CO_2_, and then washed with PBS three times. The cells were then fixed with PFA for 30 min at room temperature. The nanoparticles uptake was measured by ICP-MS as described above.

**Intracellular reactive oxygen species (ROS) generation**

For determination of ROS levels *via* fluorescent imaging, HepG2 cells were incubated for 2 h with 5 different groups: (1) PBS (2) ultrasound (US, 1.0 MHz, 1.5 W/cm^2^, 50% duty cycle, 1 min) (3) Cur (4) US+Cur (5) US+CMC . The Cur concentration was 10 μM in group 3, 4 and 5. All the cells were pre-treated with hypoxia gas. Then, the fluorescent dye, DCFH-DA (10 μM), was added and co-incubated for 20 min at 37 °C. Then, cells in group 2, 4 and 5 were irradiated with the US. ROS level was determined by confocal laser scanning microscope (CLSM; IX81, Olympus, Japan). The fluorescent intensity of each group was calculated by ImageJ software.

***In vitro* phototoxicity of CMC**

The phototoxicity was measured by MTT assay. HepG2 cells were seeded in 96-well plates at a density of 5 × 10^3^ cells per well and incubated for 24 h. Afterwards, HepG2 cells were incubated for 2 h with 5 different groups: (1) PBS (2) ultrasound (US, 1.0 MHz, 1.5 W/cm^2^, 50% duty cycle, 1 min) (3) Cur (4) US+Cur (5) US+CMC . The Cur concentration was 10 μM in group 3, 4 and 5. The normoxic conditions (O_2_) were maintained in a humidified incubator at 37℃ in 5% CO_2_ and 95% air. The hypoxic condition (N_2_) was achieved by pre-incubating the cells in a hypoxic incubator supplied with a hypoxic gas stream (1% O_2_, 5% CO_2_, and 94% N_2_) for 12h. Then, cells in group 2, 4 and 5 were irradiated with the US. At the end of the incubation, 5 mg/mL MTT PBS solution was added, and the plate was incubated for another 4 h. Finally, the absorbance values of the cells were determined by using a microplate reader (Emax Precision, USA) at 570 nm. The background absorbance of the well plate was measured and subtracted. The cytotoxicity was calculated by dividing the optical density (OD) values of treated groups (T) by the OD values of the control (C) (T/C × 100%). We then used the same method to verify the *in vitro* phototoxicity of CMC with different Cur concentration (5 and 20 μg/mL).

***In vivo* pharmacokinetics**

BALB/c mice (n = 3) received an intravenous (i.v.) injection of 100 μL PBS containing MC or CMC (with an equivalent Cur dose of 5 mg/kg). At various time points after the injection 20 μL blood plasma was collected from the tail veins and Mn^2+^ was quantitatively analyzed by ICP-MS.

***In vivo* distribution study**

When tumors reached 200mm^3^, tumor bearing mice (n = 3) received an intravenous (i.v.) injection of 100 μL PBS containing MC or CMC (with a Cur dose of 5 mg/kg). Mice were sacrificed at specific time points to collect the tumors and major organs for Mn^2+^ measurement by ICP-MS.

***In vivo* hypoxia evaluation**

The HepG2 tumor model was used. When tumors reached 200mm^3^, tumor bearing mice (n = 3) received an intravenous (i.v.) injection of 100 μL PBS containing MC or CMC (with a Cur dose of 5 mg/kg). Mice were sacrificed at 6 h after injection to collect the tumors and tumors were obtained for Pimonidazole (PIMO) staining. Specifically, PIMO positive tumor composition was analyzed using an in-house code in MATLAB.

***In vivo* antitumor study**

When tumors reached 200mm^3^, tumor bearing mice were divided randomly into 5 groups (each group included 5 mice): (1) PBS; (2) US, 1.0 MHz, 1.5 W/cm^2^, 50% duty cycle, 1 min; (3) CMC; (4) MC+US; (5) CMC+US. The Cur dose was 5 mg/kg in group 2, 3 and 4. The SDT was performed 6 h after intravenous injection. The treatment was conducted every 2 days for 14 days. Mice body weight was monitored every 2 days. After 14 days treatment, all the mice were sacrificed. Five main organs (heart, liver, spleen, lung and kidney) and tumors of all mice were harvested, washed with PBS, and fixed with paraformaldehyde for histology analysis. And the tumor tissues were imaged and weighed, and fixed in 4% neutral buffered formalin, processed routinely into paraffin, and sectioned at 4 μm. Then the sections were stained with Ki-67 and hematoxylin and eosin (H&E) and finally examined by using an optical microscope (BX51, Olympus, Japan). 

**Statistical analysis**

Data analyses were conducted using the GraphPad Prism 5.0 software. Significance between every two groups was calculated by the Student’s t-test. *P < 0.05, **P < 0.01, ***P < 0.005.


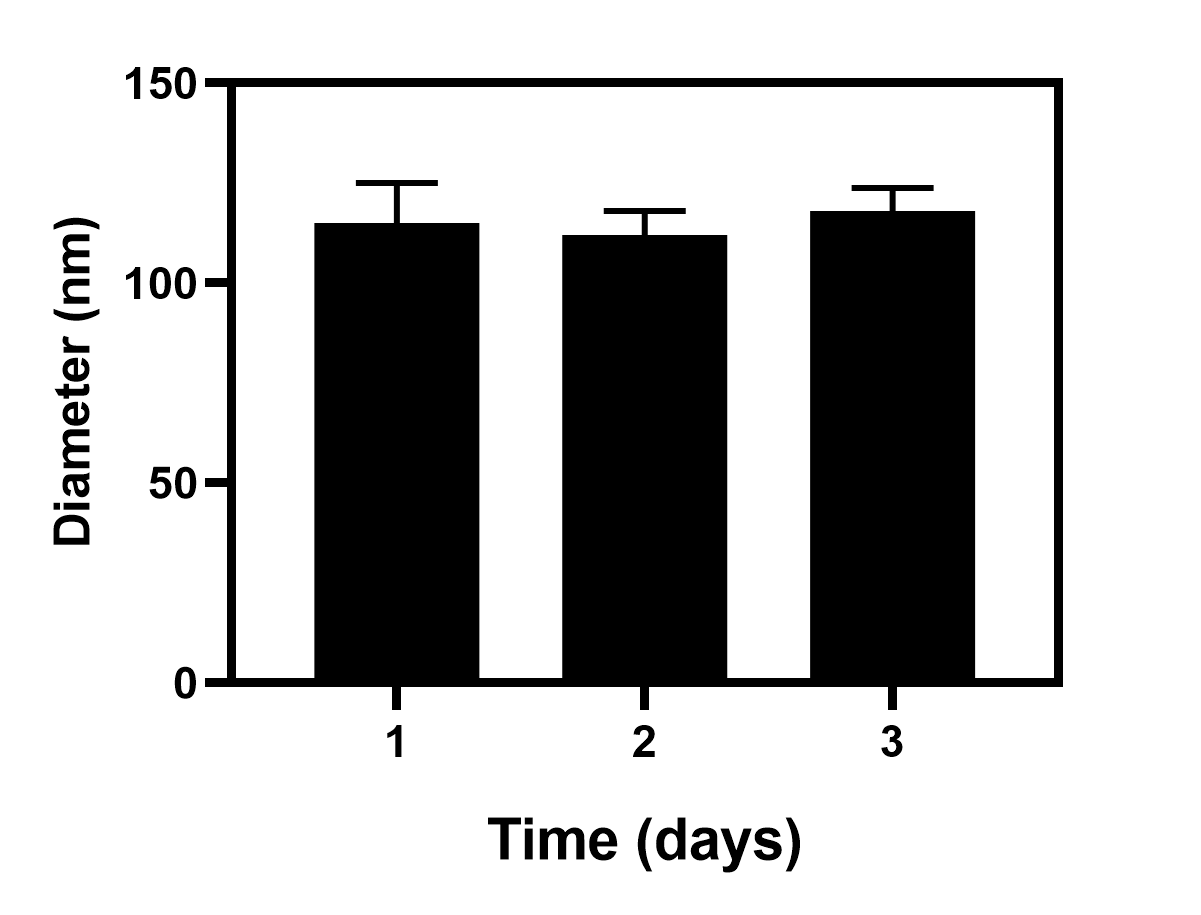


Figure S1. DLS was used to measure the hydrodynamic diameter of CMC in the FBS after 1, 2, and 3 days.

**References**

[1] G. Yang, L. Xu, Y. Chao, J. Xu, X. Sun, Y. Wu, R. Peng, Z. Liu, Hollow MnO2 as a tumor-microenvironmentresponsive biodegradable nano-platform for combination therapy favoring antitumor immune responses, Nature communications 8(1) (2017) 902.

[2] R.H. Fang, C.M. Hu, B.T. Luk, W. Gao, J.A. Copp, Y. Tai, D.E. O'Connor, L. Zhang, Cancer cell membrane-coated nanoparticles for anticancer vaccination and drug delivery, Nano letters 14(4) (2014) 2181-8.

[3] D.-M. Zhu, W. Xie, Y.-S. Xiao, M. Suo, M.-H. Zan, Q.-Q. Liao, X.-J. Hu, L.-B. Chen, B. Chen, W.-T. Wu, L.-W. Ji, H.-M. Huang, S.-S. Guo, X.-Z. Zhao, Q.-Y. Liu, W. Liu, Erythrocyte membrane-coated gold nanocages for targeted photothermal and chemical cancer therapy, Nanotechnology 29(8) (2018) 084002.

[4] R.H. Fang, A.V. Kroll, W. Gao, L. Zhang, Cell Membrane Coating Nanotechnology, Advanced materials 30(23) (2018) e1706759.

[5] X. Yang, Y. Yang, F. Gao, J.-J. Wei, C.-G. Qian, M.-J. Sun, Biomimetic Hybrid Nanozymes with Self-Supplied H+ and Accelerated O2 Generation for Enhanced Starvation and Photodynamic Therapy against Hypoxic Tumors, Nano letters 19(7) (2019) 4334-4342.
